# *BRAF*突变型非小细胞肺癌免疫检查点抑制剂治疗进展

**DOI:** 10.3779/j.issn.1009-3419.2019.09.06

**Published:** 2019-09-20

**Authors:** 夏 刘, 殿胜 钟

**Affiliations:** 300052 天津，天津医科大学总医院肿瘤内科 Department of Medical Oncology, Tianjin Medical University General Hospital, Tianjin 300052, China

**Keywords:** 肺肿瘤, 鼠类肉瘤病毒癌基因同源物B1, 免疫检查点抑制剂治疗, Lung neoplasms, V-raf murine sar-coma viral oncogene homolog B1, Immune checkpoint inhibitor therapy

## Abstract

在非小细胞肺癌（non-small cell lung cancer, NSCLC）患者中，约2%-4%有*BRAF*基因突变，该型肿瘤恶性程度高、化疗有效率低、预后差。尽管BRAF抑制剂及MEK抑制剂联合治疗在*BRAF* V600E突变的晚期NSCLC患者中成效显著，已被写入美国国家综合癌症网络（National Comprehensive Cancer Network, NCCN）指南，但两药联合副作用发生率高，耐药之后尚无有效治疗策略，且针对非V600E突变患者仍缺乏靶向治疗方案。本文将针对*BRAF*突变型NSCLC免疫标志物表达情况以及免疫检查点抑制剂（immune checkpoint inhibitor, ICI）疗效相关研究做一综述，为延长患者生存提供更多选择方案。

## Abstract (en)

*BRAF* gene mutation is found in about 2%-4% of the patients with non-small cell lung cancer (NSCLC). This type of NSCLC is characterized by high malignancy, low efficacy of chemotherapy and poor prognosis. Although the combination treatment of BRAF inhibitor and MEK inhibitor has achieved remarkable results in advanced NSCLC patients with *BRAF* V600E mutation, which has been written into the National Comprehensive Cancer Network (NCCN) guidelines, severe side effects of the combination therapy are frequently observed. There isn't effective treatment strategy after drug resistance, and targeted therapy for non-V600E mutation patients is still lacking. In this paper, we summarized the researches on expression of immune markers in NSCLC patients with mutant BRAF and analyzed the studies on efficacy of immune checkpoint inhibitor (ICI), so as to provide more options for prolonging survival of the patients.

随着靶向药物及免疫检查点抑制剂（immune checkpoint inhibitor, ICI）的飞速进步，晚期非小细胞肺癌（non-small cell lung cancer, NSCLC）的治疗发生了巨大变化，根据有无驱动基因来决定一线治疗已成为临床医生的重要决策。多项Ⅲ期研究证明，伴有表皮生长因子受体（epidermal growth factor receptor, *EGFR*）、*BRAF*突变或*ALK*、*ROS1*基因融合患者，一线接受TKI治疗较传统化疗的客观缓解率（objective response rate, ORR）及无进展生存期（progression free survival, PFS）显著提高。在驱动基因阴性的NSCLC中，当程序性细胞死亡蛋白-配体1（programmed cell death protein-ligand 1, PD-L1）表达≥50%时，ICI治疗较化疗显著提高中位生存期（overall survival, OS）^[1]^；当PD-L1表达 < 50%，多项研究^[2, 3]^也显示，化疗联合ICI治疗较单纯化疗有生存优势。

*BRAF*突变率占NSCLC的2%-4%，主要为腺癌；V600E突变约占全部*BRAF*突变病例的50%。*BRAF* V600E突变晚期患者应用BRAF抑制剂治疗ORR为33%-42%，中位PFS为5.5个月-7.3个月；应用BRAF抑制剂和MEK抑制剂联合治疗，ORR达63%-64%，中位PFS达9.7个月-10.9个月^[4]^。然而，靶向治疗严重不良反应发生率为42%-56%，且大部分患者不可避免产生耐药，OS数据还不明确；针对*BRAF*非V600E突变NSCLC，尚缺乏行之有效的靶向治疗方案，故探索更加卓有成效的治疗策略势在必行。ICI治疗用于驱动基因突变型NSCLC患者的数据有限，ICI对*BRAF*突变型NSCLC的疗效尚不清楚，既往NSCLC患者接受抗程序性细胞死亡蛋白-1（programmed cell death protein-1, PD-1）/抗PD-L1药物的大型临床试验也很少报告这一亚组的结果。*BRAF*突变型NSCLC是否和EGFR/ALK阳性患者一样，很难从ICI治疗中获益^[5, 6]^呢？本文将就*BRAF*突变型NSCLC患者PD-L1表达情况、肿瘤突变负荷（tumor mutational burden, TMB）和微卫星不稳定性（microsatellite instability, MSI）状态，以及ICI治疗的疗效等做一梳理。

## 1 *BRAF*突变型NSCLC免疫相关标志物检测

*BRAF*突变型NSCLC免疫标志物的相关研究多为回顾性，且因*BRAF*突变几率较低，均为小样本研究。Yang等^[7]^分析了163例手术切除的Ⅰ期肺腺癌患者PD-L1表达与*EGFR*、*KRAS*、*BRAF*、*ALK*等基因突变的关系，采用巢式PCR检测*BRAF* 15外显子突变情况。结果显示，总体人群的PD-L1阳性率为39.9%（65/163）；*BRAF*突变7例，其中4例（57.1%）PD-L1阳性，虽高于总体人群PD-L1表达率，但由于数据量小，未观察到BRAF与PD-L1的相关性，*BRAF*突变与5年PFS及5年OS也无显著相关性。随后，Yang^[8]^等又对105例手术切除的Ⅰ期肺鳞癌患者PD-L1表达与*EGFR*、*KRAS*、*BRAF*、*ALK*、*PI3KCA*和*FGFR1*等基因突变的关系进行分析，总体人群的PD-L1阳性率为56.2%（59/105），与Ⅰ期肺鳞癌良好的免疫微环境及临床疗效密切相关。*BRAF*突变患者8例，未检测到*BRAF* V600E突变，其中2例突变伴随PD-L1阳性表达，但未观察到*BRAF*突变与PD-L1表达的相关性。Song等^[9]^分析了385例Ⅰ期-Ⅲ期手术切除肺腺癌患者中PD-L1与*EGFR*、*ALK*、*KRAS*、*HER2*、*BRAF*等基因改变的关系，PD-L1阳性表达率为48.3%（186/385），*BRAF*突变患者2例（0.5%），均为PD-L1阳性表达（100%）。以上三项均为回顾性研究，样本量相对较小，*BRAF*突变率较低，即使进行合并分析^[10]^，仍未观察到PD-L1与*BRAF*突变的相关性。

Tseng^[11]^也对211例Ⅰ期-Ⅳ期NSCLC患者PD-L1表达与*BRAF*突变的相关性进行回顾性分析，经过对PD-L1及*EGFR*、*ALK*、*HER2*、*BRAF* V600E检测，发现3例*BRAF* V600E突变患者。总体人群PD-L1阳性率（≥1%）及强阳性率（≥50%）分别为27.0%和12.8%，与吸烟程度呈正相关；2例*BRAF* V600E突变患者伴有PD-L1强阳性表达（66.7%）；*EGFR*、*ALK*、*HER2*和*BRAF* V600E突变的不吸烟者均无PD-L1强阳性表达。在接受PD-1检查点抑制剂治疗的患者中，PD-L1高表达与更长的PFS有关[HR=0.15, 95%CI: 0.03-0.71, *P*=0.017]。研究提示，PD-L1状态是影响ICI治疗效果的重要指标，无论何种突变，吸烟患者更可能伴有较强的PD-L1表达。由于病例数量有限，需要进一步的研究来验证结果。

以上均为亚裔数据，Mufti^[12]^近期报告了2例*BRAF*突变合并PD-L1阳性非亚裔病例的个案分析。2例患者均以大量心包积液为首发症状，且病理类型均为腺癌，1例否认吸烟史，有*BRAF* V600E突变合并PD-L1表达；另1例有重度吸烟史，突变类型为*BRAF* D594N，均正在接受达拉非尼联合曲美替尼治疗。

Dudnik等^[13]^学者发表了第一个在*BRAF*突变型NSCLC患者中观察PD-L1表达及ICI疗效的研究，也是目前为止规模最大的BRAF突变型NSCLC接受ICI治疗的研究。研究者从7家癌症中心纳入*BRAF*突变型NSCLC 39例，绝大多数（37/39）为晚期肺癌。其中，*BRAF* V600E突变21例（54%），*BRAF*非V600E突变18例（46%）。患者中位年龄66岁，女性17例（44%），不吸烟15例（38%）。研究者分别对29例（74%）、11例（28%）和12例（31%）患者进行了PD-L1表达、TMB和MSI分析。在*BRAF* V600E突变的19例患者中，6例PD-L1表达1%-49%，8例PD-L1表达≥50%。10例非V600E *BRAF*突变患者，PD-L1表达1%-49%有1例（10%），PD-L1表达≥50%为5例（50%）。进一步分析显示，中位TMB为7 muts/mb（范围为1-42），18%肿瘤的TMB≥20 muts/mb；在8例*BRAF* V600E突变患者中，2例TMB较高（25%），在非V600E突变患者中未发现高TMB或MSI；所有患者的肿瘤都没有发生MSI。

在2018年美国临床肿瘤学会（American Society of Clinical Oncology, ASCO）会议上，Mazieres汇报了一项回顾性多中心研究--ImmunoTarget，探讨驱动基因阳性晚期NSCLC患者接受ICI治疗的疗效。研究共纳入25个中心的551例Ⅳ期肺癌患者，驱动基因变异包括*KRAS*（*n*=271）、*EGFR*（*n*=125）、*BRAF*（*n*=43）、*MET*（*n*=36）、*HER2*、*ALK*、*RET*、*ROS1*等，大部分患者接受二线ICI治疗。*BRAF*突变患者的中位年龄61岁，吸烟者占比72%，V600E突变占比48%；10例患者进行了PD-L1检测，其中7例为阳性表达（70%）。

总的来说，*BRAF*突变型NSCLC常见于吸烟、男性患者；且PD-L1表达水平明显增高，既与*EGFR*/*ALK*基因改变患者临床特点不同^[14]^，也高于未选择NSCLC患者^[15]^，提示NSCLC中*BRAF*突变与PD-L1高表达可能相关。TMB与非选择NSCLC患者群体数据基本一致^[16]^。这些有待更大规模研究进一步分析。

## 2 *BRAF*突变型NSCLC的ICI疗效研究

在Dudnik的研究中^[13]^，共有22例晚期*BRAF*突变患者接受抗PD-1/PD-L1治疗。针对20例疗效可评估患者，ORR为28%，V600E突变组和非V600E突变组的ORR分别为25%和33%（*P*=1.0）。ORR在PD-L1表达≥50%的亚组为36%，在PD-L1表达 < 50%的亚组为14%（*P*=0.59）。*BRAF* V600E突变且PD-L1表达≥50%的8例患者中，3例一线接受pembrolizumab治疗，ORR为33%。*BRAF* V600E突变亚组与非V600E突变亚组患者的中位PFS分别为3.7个月（95%CI: 1.6-6.6）和4.1个月（95%CI: 0.1-19.6）（*P*=0.37）；PD-L1表达≥50%者及1%-49%者的中位PFS分别为5.3个月（95%CI: 1.7-6.6）和2.2个月（95% CI: 0.1-8.2，*P*=0.73）。*BRAF*突变类型及PD-L1表达与ICI治疗的ORR及PFS均没有显著相关性。接受ICI治疗患者的中位OS虽未达到（95%CI: 13-NR），但显著高于未接受ICI治疗的21.1个月（95%CI: 1.8-NR, *P*=0.018）。总的来说，*BRAF*突变型NSCLC患者接受ICI治疗的ORR为25%-33%，PFS为3.7个月-4.1个月，与非选择NSCLC人群二线治疗疗效相似，高于EGFR阳性及MET外显子14改变的NSCLC患者^[15, 17-19]^。ICI治疗有效，这在驱动基因阳性患者ICI治疗的临床研究中是很难见到的，可能与*BRAF*突变型NSCLC中吸烟者较多、PD-L1阳性表达较高及相对较高的TMB有关。研究未对所有患者常规进行BRAF检测，并且PD-L1检测仅在74%的患者中进行，TMB和MSI状态仅在30%左右病人中评估，可能引起选择偏倚，并且只有一半患者接受了ICI治疗，缺乏统一的疗效评估间隔，导致ICI治疗的疗效评估不确切，故需要在更大规模前瞻性研究中验证。

Mazieres在2018年ASCO会议报道了*KRAS*、*EGFR*、*BRAF*等多个基因突变晚期NSCLC患者接受ICI治疗的疗效，总体人群的ORR为19%（15-22%），中位PFS为2.8个月（2.5-3.1）；中位OS为13.3个月（9.8-14.8）。除*EGFR*突变患者，总体人群的PFS与吸烟（*P*=0.003）及PD-L1表达（*P*=0.02）呈正相关。*KRAS*和*BRAF*突变的患者较*EGFR*、*ALK*和*RET*变异患者接受ICI治疗的疗效更好。*BRAF*突变患者的ORR为24%，仅次于*KRAS*突变患者的28%，高于其他突变亚型，mPFS为3.1个月，mOS为13.6个月。*BRAF*突变吸烟患者的PFS明显优于不吸烟患者（*P*=0.03），但*BRAF* V600E突变与其他类型*BRAF*突变的PFS无显著差异，在*BRAF*突变患者中也未观察到疗效与PD-L1表达的相关性。2018年世界肺癌大会（World Conference on Lung Cancer, WCLC）会议上，研究者依据*BRAF*突变类型对于ICI治疗的最佳疗效进一步分析，具有可评估疗效的13例V600E突变患者，ORR为15.4%，疾病控制率（disease control rate, DCR）为38.5%，17例非V600E突变患者的疗效优于V600E突变患者，ORR为41.2%，DCR达到64.7%。*BRAF*突变患者的ORR与吸烟状态及PD-L1表达无明显相关。该研究为回顾性研究，且各亚组人数较少，故应谨慎解读其结果。

近期在JTO发表的真实世界的研究^[20]^，对入组意大利Expanded Access Program（EAP）二线接受nivolumab治疗且进行过BRAF检测的210例晚期非鳞NSCLC患者进行分析，其中*BRAF*突变患者11例（5%）。这项研究虽然是回顾性分析、患者例数较少、病人组间不平衡，但是OS在ICI治疗的不同亚组间是略有区别的：BRAF状态未知人群的OS为11.0个月（9.8-12.2），BRAF野生型和*BRAF*突变人群的OS分别为11.2个月（9.2-13.2）和10.3个月（2.1-18.5）。11例*BRAF*突变患者中仅有1例77岁男性患者（*BRAF* V600E突变，腺癌，有吸烟史）达到部分缓解（partial response, PR）并维持29个月。ORR仅为9%，略低于Dudnik及Mazieres等的数据，但与野生型NSCLC二线ICI治疗疗效相近。遗憾的是，PD-L1和TMB没有在该研究中进行常规评估，但其结论支持进一步深入分析。

综上，ICI对于*BRAF*突变型NSCLC可能具有一定的治疗价值。TMB低、PD-L1表达水平低、不吸烟及与部分驱动基因突变（例如，*EGFR*基因突变）已被确定可能是ICI疗效较差的特征人群。ICI治疗效果在*BRAF*突变型NSCLC中比较突出可能与吸烟者比例更高、PD-L1和TMB相对高表达有关。这些数据支持*BRAF* V600E突变NSCLC在BRAF TKI疗效不好时，尝试ICI治疗。由于*BRAF* V600E突变与其他类型*BRAF*突变的PFS无显著差异，针对BRAF非V600E突变NSCLC，ICI治疗可能是优于靶向治疗的选择。

## 3 *BRAF*突变型NSCLC应用ICI治疗的机制

*BRAF*突变NSCLC应用ICI治疗相关机制文献较少，可借鉴黑色素瘤的研究。大约40%-50%的转移性皮肤黑色素瘤患者存在*BRAF* V600突变，肿瘤细胞PD-1、PD-L1表达及MSI-H均为ICI治疗的选择依据。TMB及相应的新抗原表达^[21]^患者对ICI的临床反应更好，提示突变相关肿瘤抗原特异性T细胞在促进肿瘤消退中起着重要作用。针对细胞毒性T淋巴细胞抗原-4（cytotoxic T lymphocyte antigen-4, CTLA-4）、PD-1及PD-L1的单克隆抗体在黑色素瘤中具有较高的活性。Ipilimumab是FDA批准的第一个抗CTLA-4药物，Nivolumab和Ipilimumab联合治疗初治患者比单独使用Nivolumab或Ipilimumab疗效更好，然而疗效与BRAF状态无关^[22, 23]^。临床实践证实TMB高的黑色素瘤患者较TMB低的患者更能够从CTLA4/PD-1抑制剂治疗中获益。*BRAF*、*NRAS*、*NF1*是黑色素瘤中常见的三种驱动基因突变，三种突变的TMB分别为12/MB、18/MB、63/MB。目前的研究表明，*NRAS/NF1*突变与ICI疗效正相关^[24]^，而BRAF则与ICI的疗效不相关^[25]^。关于*BRAF*突变状态与黑色素瘤ICI疗效及生物标志物的研究仍在进行^[22]^。

关于*BRAF*突变NSCLC应用ICI治疗的机制，Sminth^[26]^报道的个案也许能够带来一些启示。这是1例76岁老年女性，2012年接受右下肺叶切除术，分期为T3N0M0，病理诊断为高中分化腺癌。患者完成辅助化疗之后9个月，出现肺部新发结节，活检证实腺癌复发。二线接受单药Nivolumab治疗，7个月后转移瘤完全消失，目前缓解持续时间已经达到4.5年。患者的全外显子测序显示肿瘤有30个非同义外显子突变，同时存在*BRAF* N581I突变，且ALK、EGFR、ROS 1和KRAS均为阴性，肿瘤细胞PD-L1表达阴性，血管周围淋巴细胞聚集且PD-L1表达阳性，免疫表型证实了CD8^+ ^T淋巴细胞的存在。Sminth团队用MANAFEST法^[27]^检测了T细胞对突变相关新抗原的反应。患者在接受抗PD-1治疗2年后，26个候选新抗原中有23个诱导了CD8^+ ^T细胞的显著性、特异性、克隆性扩增，其中2个肿瘤抗原含致癌基因*BRAF* N581I突变，这是黑色素瘤和结直肠癌中经常发生的热点突变^[28]^。*BRAF* N581I与*BRAF* V600E突变的致癌机制不同，N581I降低或不激活BRAF激酶活性，但可诱导KRAS依赖的CRAF信号转导和ERK激活^[29]^。值得关注的是，T细胞对*BRAF* N581I和其他22个突变相关肿瘤抗原的识别，在肿瘤完全消退数年后仍持续存在，从而定义了一种抗肿瘤记忆T细胞反应。驱动突变可以激发长效内源性抗肿瘤免疫反应，从而可能促进接受ICI治疗的患者的临床反应。在这一点上，热点驱动基因突变所特有的T细胞过继转移，包括HLAI-限制性KRAS G12D表位特异性CD8^+ ^T细胞^[30]^和HLAII-限制性*BRAF* V599E^[31]^或*BRAF* V600E^[32]^突变特异性CD4^+ ^T细胞，已被证实具有临床价值。因此，若想达到长期肿瘤控制，针对驱动突变的持续性T细胞反应可能更有效。

## 4 *BRAF*突变型NSCLC首先选择ICI治疗还是靶向治疗

*BRAF*突变NSCLC的ICI治疗刚刚起步，未查到一线治疗选择的研究报道，但从黑色素瘤的研究可以找到一些线索。在转移性黑色素瘤中，尚缺乏免疫检查点抑制剂和靶向治疗之间的面对面比较^[33]^，最佳一线治疗顺序的问题仍然没有得到明确解答，可参考以下研究。Yao^[34]^回顾性分析了1, 140例2014年-2016年接受一线治疗的晚期黑色素瘤患者，归纳总结最常见的一线治疗方案比例，分别为Ipilimumab（34%）、抗PD-1单药（26%）和BRAF/MEK抑制剂（20%），由此可见ICI治疗比例明显高于靶向治疗。Ghate^[35]^回顾分析了两个大型的美国商业索赔数据库中，2014年-2016年转移性黑色素瘤的一、二线治疗模式，结果显示，一线接受靶向治疗的比例为62%-66%，二线靶向治疗比例为52%-54%，与上一个研究有所差异。在Pembrolizumab对比Ipilimumab的关键研究中^[36]^，既往接受过BRAF抑制剂治疗的患者接受ICI治疗同未接受BRAF抑制剂治疗的获益一致。在随后Nivolumab联合Ipilimumab对比单药Nivolumab或ipilimumab的研究中^[37]^，*BRAF* V600E突变亚组ICI联合治疗较BRAF野生型亚组有OS优势（HR 0.69 *vs* 0.94），提示高强度ICI治疗在*BRAF*突变亚组更获益，而Nivolumab治疗*BRAF*突变和野生型的OS一致。这些接受CTLA-4和PD-1抑制剂的单药或联合治疗的经验提示，ICI治疗的疗效不受BRAF/MEK抑制剂的影响，*BRAF* V600E突变亚组ICI联合治疗疗效更好。目前黑色瘤普遍的共识是^[38-40]^，对于有症状、重要器官受累或肿瘤负荷较大的患者建议接受ICI治疗前先给予BRAF/MEK抑制剂，以达到肿瘤快速缩小、改善症状的目的；鉴于PD-1抑制剂良好的安全性，在不优先考虑快速反应的情况下，支持将其作为一线治疗。

在*BRAF* V600E突变NSCLC中，达拉菲尼联合曲美替尼的有效率较高，但是毒性值得关注（特别是发热、胃肠功能紊乱），而单药抗PD-1/PD-L1抑制剂总体耐受性较好。*BRAF*突变型NSCLC患者年龄大，既往多伴有吸烟史，如果患者没有肿瘤高负荷，不需要快速缩瘤减轻症状，可尝试首先给予ICI治疗。BRAF/MEK抑制剂治疗的*BRAF* V600E突变疗效不受治疗线数的影响^[41, 42]^，可保留为后续的治疗手段。

Li^[43]^报道了1例74岁女性、既往吸烟的转移性肺腺癌病例，一线、二线分别应用培美曲塞联合索拉非尼以及培美曲塞联合卡铂，由于不良反应停药。FoundationOne基因组分析证实患者存在*BRAF* V600E突变以及ATM等肿瘤抑制因子的失活突变，另外患者TMB为5/Mb，MSS，但PD-L1高表达，为90%。患者接受了19个月的达拉非尼治疗直至疾病进展，随后患者接受了2个周期Pembrolizumab治疗，正电子放射断层扫描（positron emission tomography-computer tomography, PET-CT）提示治疗有效，但由于免疫性结肠炎及肺炎停药。患者从诊断晚期肿瘤至末次随访，生存期超过7年，目前没有症状。对BRAF V600E和PD-L1同时阳性的NSCLC患者进行ICI治疗之前，首先使用BRAF抑制剂也是合理的，前提是副作用可以耐受。鉴于*BRAF*突变在NSCLC中的比例较低，进行有关治疗顺序相关的前瞻性研究难度大，临床医生需要综合临床研究数据和患者状态来决定。

## 5 *BRAF*突变型NSCLC靶向联合免疫治疗的可能性

2019年美国国立综合癌症网络（National Comprehensive Cancer Network, NCCN）黑色素瘤指南推荐nivolumab联合CTLA-4抗体ipilimumab（I-O联合）一线治疗BRAF野生型晚期皮肤黑色素瘤患者，使晚期皮肤黑色素瘤3年OS达到58%^[37]^。进展缓慢、无症状的*BRAF* V600突变型患者也可首选上述治疗方案。免疫联合的机制包括：促进抗原递呈，触发免疫应答；改善肿瘤免疫微环境，促进淋巴细胞浸润肿瘤组织等。另有研究显示，RAS-RAF-MAP通路异常活化后，可促进肿瘤细胞PD-L1表达、抑制人类白细胞抗原（human leukocyte antigen, HLA）I类分子表达和抑制T细胞浸润肿瘤组织。RAF和MEK抑制剂可部分纠正MAPK信号的免疫抑制效应。例如，2016年黑色素瘤研究学会（Society for Melanoma Research, SMR）会议报道，PD-L1抗体Atezolizumab、BRAF V600E抑制剂Vemurafenib联合MEK抑制剂Cobimetinib治疗34例*BRAF* V600突变的晚期黑色素瘤，有效率为85%。因此，靶向药物联合免疫治疗（ICI、细胞因子治疗、过继性细胞免疫治疗等）很可能会产生协同增敏作用，发挥早期而强效的抑瘤功能，获得更好的临床疗效，这在晚期黑色素瘤治疗中是一个选择，更多相关研究正在逐步展开。在*BRAF*突变NSCLC领域，我们也期待有更多靶向联合免疫、免疫联合免疫的基础及临床研究，为患者带来生存获益。

## 6 小结

*BRAF*突变虽在NSCLC中发生比例较低，但是突变患者易转移、预后差、治疗手段有限。随着靶向治疗的不断深入，晚期患者的预后得以改善，但副作用明显，耐药不可避免。本文中研究提示ICI治疗对*BRAF*突变型NSCLC的疗效优于其他驱动基因突变，与*BRAF*突变类型无关，且安全性较好，可作为靶向治疗之外的选择策略。由于进行前瞻性研究难度较大，治疗方案、顺序选择等问题还需临床医生综合临床研究数据和患者状态来决定。期待多中心的关注、协作和共同研究，为*BRAF*突变晚期NSCLC患者的靶向、免疫及联合治疗提供更可靠的理论依据和技术支持。
